# Effect of Iron Overload and Iron Deficiency on Liver Hemojuvelin Protein

**DOI:** 10.1371/journal.pone.0037391

**Published:** 2012-05-18

**Authors:** Jan Krijt, Jana Frýdlová, Lenka Kukačková, Yuzo Fujikura, Petr Přikryl, Martin Vokurka, Emanuel Nečas

**Affiliations:** Institute of Pathophysiology, Charles University in Prague, First Faculty of Medicine, Prague, Czech Republic; University of Leuven, Belgium

## Abstract

**Introduction:**

Hemojuvelin (Hjv) is a key component of the signaling cascade that regulates liver hepcidin (Hamp) expression. The purpose of this study was to determine Hjv protein levels in mice and rats subjected to iron overload and iron deficiency.

**Methods:**

C57BL/6 mice were injected with iron (200 mg/kg); iron deficiency was induced by feeding of an iron-deficient diet, or by repeated phlebotomies. Erythropoietin (EPO)-treated mice were administered recombinant EPO at 50 U/mouse. Wistar rats were injected with iron (1200 mg/kg), or fed an iron-deficient diet. Hjv protein was determined by immunoblotting, liver samples from *Hjv*−/− mice were used as negative controls. Mouse plasma Hjv content was determined by a commercial ELISA kit.

**Results:**

Liver crude membrane fraction from both mice and rats displayed a major Hjv-specific band at 35 kDa, and a weaker band of 20 kDa. In mice, the intensity of these bands was not changed following iron injection, repeated bleeding, low iron diet or EPO administration. No change in liver crude membrane Hjv protein was observed in iron-treated or iron-deficient rats. ELISA assay for mouse plasma Hjv did not show significant difference between *Hjv*+/+ and *Hjv−/−* mice. Liver *Hamp* mRNA, *Bmp6* mRNA and *Id1* mRNA displayed the expected response to iron overload and iron deficiency. EPO treatment decreased *Id1* mRNA, suggesting possible participation of the bone morphogenetic protein pathway in EPO-mediated downregulation of *Hamp* mRNA.

**Discussion:**

Since no differences between Hjv protein levels were found following various experimental manipulations of body iron status, the results indicate that, *in vivo*, substantial changes in *Hamp* mRNA can occur without noticeable changes of membrane hemojuvelin content. Therefore, modulation of hemojuvelin protein content apparently does not represent the limiting step in the control of *Hamp* gene expression.

## Introduction

Humans lack a regulated pathway for iron excretion, and body iron stores are therefore determined solely at the level of iron uptake in the small intestine. Mammalian iron absorption is primarily controlled by the hepatocyte-derived peptide hepcidin, encoded by the *HAMP* gene. Hepcidin regulates ferroportin-mediated iron export from enterocytes and macrophages, and thus determines dietary iron uptake, as well as the amount of iron available for erythropoiesis [1], [2], [3], [4].

Hepcidin expression is in turn regulated by three key factors: body iron stores, inflammation, and erythropoietic activity. In mice, iron overload or inflammation increase *Hamp* mRNA, while iron deficiency or accelerated erythropoiesis decrease it. At present, the signaling pathway responsible for inflammation-mediated activation of *Hamp* gene transcription has to a significant extent been elucidated, and substantial progress has been made towards the understanding of *Hamp* gene regulation by iron overload and iron deficiency. On the other hand, the mechanisms responsible for *Hamp* mRNA downregulation by accelerated erythropoiesis are still largely unknown [2], [3].

An important part of the processes related to the transcriptional control of hepcidin biosynthesis occurs at the hepatocyte plasma membrane. The key signaling pathway which regulates *Hamp* gene expression in response to liver iron stores is the bone morphogenetic protein/hemojuvelin (Bmp/Hjv) pathway [5], [6], [7], whose central component is the glycosylphosphatidylinositol (GPI)-anchored protein hemojuvelin (Hjv), encoded by the *Hfe2* gene [8]. The first known step in the signaling cascade which ultimately leads to hepcidin secretion by the hepatocyte is the transcriptional activation of bone morphogenetic protein 6 (Bmp6) expression [9]. Bmp6, probably a product of non-parenchymal liver cells [10], subsequently binds to Hjv at the extracellular side of the hepatocyte membrane, resulting in effective interaction of Bmp6 with its transmembrane receptors. Hjv thus serves as a Bmp6 coreceptor. Activation of Bmp receptors then results in intracellular phosphorylation of Smad proteins, and finally in transcriptional activation of hepcidin synthesis. The importance of Bmp6 and hemojuvelin in hepcidin expression is evident from the fact that disruption of mouse *Bmp6* [6], [7] or *Hfe2* [11], [12] genes results in severe hemochromatosis, which is in terms of tissue iron overload similar to hemochromatosis caused by disruption of the hepcidin gene itself. At present, the Bmp/Hjv pathway is therefore regarded as the main pathway which controls *Hamp* expression in response to liver iron levels. In addition to *Hamp*, the Bmp/Hjv pathway also activates transcription of several other genes, of which the *Id1* gene is often used as a convenient marker of Bmp6-dependent signaling [9].

A less severe form of hemochromatosis, associated with a less dramatic decrease in hepcidin expression, is caused by disrupted synthesis of two other membrane proteins: transferrin receptor 2 (Tfr2) [13] and the Hfe protein [14]. The exact role of these two proteins in hepcidin gene regulation is unknown, but they very probably participate in the sensing of plasma iron levels [15].

The most recently identified hepatocyte membrane protein involved in iron homeostasis regulation is the serine protease matriptase-2, encoded by the *Tmprss6* gene [16], [17], [18]. In contrast to Bmp6, Hjv, Tfr2 and Hfe, which increase hepcidin transcription, matriptase-2 is a negative regulator of hepcidin. Mutations in *TMPRSS6* cause inappropriately high hepcidin synthesis, resulting in iron-refractory iron deficiency anemia in humans. Mice with disrupted function of the matriptase-2 protein display high *Hamp* mRNA in the face of decreased liver iron stores and iron-deficient erythropoiesis [16], [18], [19], as well as a blunted response to erythropoietin (EPO) administration [20], [21]. Evidently, functional matriptase-2 is indispensable in order to adequately lower hepcidin transcription in response to physiological stimuli.

Since matriptase-2 is a membrane-bound serine protease, it very probably acts by cleaving an essential component of the hepcidin-regulatory pathway. By extensive *in vitro* experiments, Silvestri *et al.* have identified membrane hemojuvelin as the main target of matriptase-2 proteolytic activity [22]. By cleaving hemojuvelin, matriptase-2 is proposed to remove a crucial constituent of the Bmp/Hjv pathway from the membrane, and thus decrease *Hamp* expression. This concept has been further strengthened by studies on double mutant mice lacking both matriptase-2 and hemojuvelin [23], [24], which display a phenotype similar to hemojuvelin-mutant mice, as well as by the demonstration that, in cultured hepatocytes, hypoxia increases matriptase-2 expression and decreases membrane Hjv protein [25]. Recently, it has been also reported that iron-deficient diet increases matriptase-2 protein in rats [10]. Based on all these data, the emerging model of *Hamp* gene regulation by iron deficiency or hypoxia links increased matriptase-2 enzymatic activity to a decrease of Hjv protein at the hepatocyte plasma membrane [4], [10], [25].

Despite the fact that hemojuvelin represents an important component in the regulation of hepcidin expression, there is comparatively little information on liver hemojuvelin protein content *in vivo*. Particularly, reliable data from mouse models are lacking, mainly because most antibodies detect false positive bands on immunoblots [23]. Recently, we have used mice with targeted disruption of the hemojuvelin gene (*Hjv*−/− mice) to identify Hjv-specific bands on immunoblots, and to optimize conditions for hemojuvelin detection with a commercial antibody [26]. The primary aim of the present study was to determine hemojuvelin expression in states of altered iron homeostasis. The obtained results show that hemojuvelin protein levels were not altered by iron deficiency or iron overload, indicating that substantial variation in *Hamp* gene transcription can occur without noticeable changes in membrane hemojuvelin content.

## Results

### Manipulation of iron levels does not change membrane hemojuvelin content in mice

**Figure 1:**
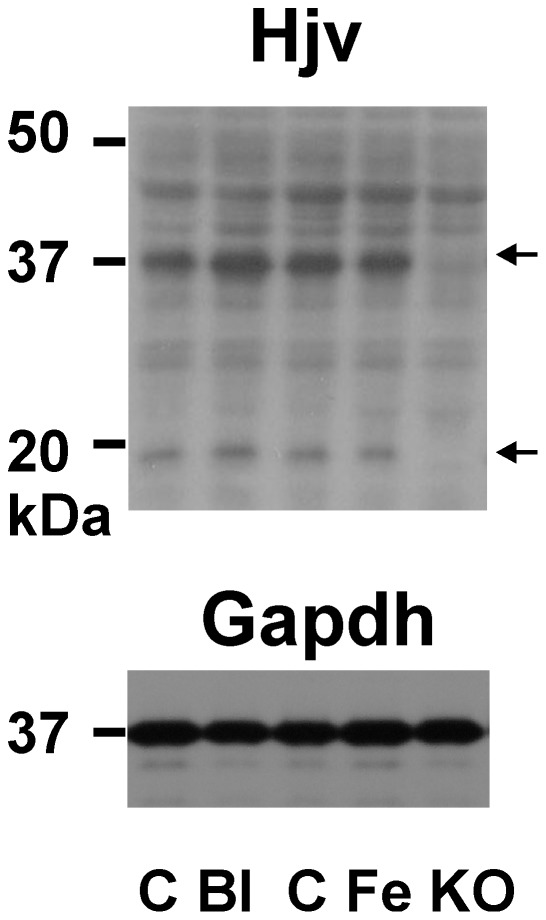
Hemojuvelin protein in mouse whole liver homogenates. C: Control, Bl: Bleeding (0.6 ml of blood once weekly for 3 weeks), Fe: Iron injection (200 mg/kg), KO: *Hjv*−/− mice. 80 µg of protein was loaded per lane. Arrows indicate Hjv-specific bands. Gapdh was used as loading control. Primary anti-Hjv antibody: AF3634.

Immunobloting of mouse Hjv is generally difficult due to the presence of non-specific bands [20], [23]. Therefore, to identify hemojuvelin-specific bands on immunoblots, we have previously used whole liver lysates from *Hjv−/−* mice as negative controls [26]. This approach enabled selection of a suitable commercial antibody (AF3634), which, under reducing conditions, detects two hemojuvelin-specific bands at approximately 35 and 20 kDa (Fig. 1). These bands very probably represent two components of Hjv heterodimer, which forms subsequently to autoproteolytic cleavage of the full-length Hjv monomer [27], [28]. Detection of the 35 kDa band was highly reproducible; on the other hand, the signal from the 20 kDa band was weaker and its relative intensity varied with the individual batch of the antibody used.

**Figure 2:**
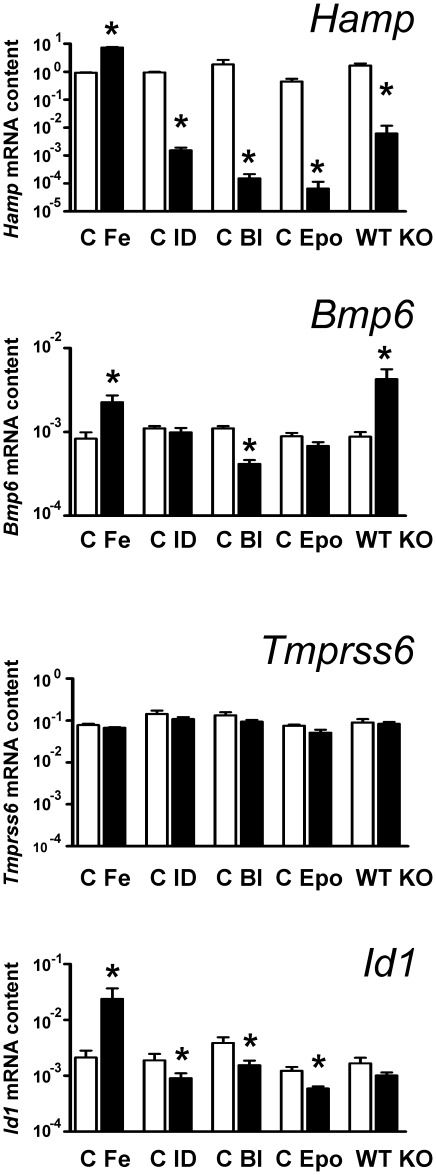
Effect of iron status on mouse liver *Hamp*, *Bmp6*, *Tmprss6* and *Id1* mRNA. *Hamp*, *Bmp6*, *Tmprss6* and *Id1* mRNA was determined by real-time PCR and expressed relative to β-actin mRNA. C: Control, Fe: Iron injection (200 mg/kg), ID: Iron-deficient diet, Bl: Bleeding (0.6 ml of blood once weekly for 3 weeks), Epo: Erythropoietin (50 U/day) administration for 4 days, WT: *Hjv*+/+ mice, KO: *Hjv*−/− mice. Asterisks indicate statistically significant difference (n = 4, p<0.05).

Comparison of hemojuvelin-specific bands in whole liver homogenates from C57BL/6 mice subjected to iron overload or iron deficiency showed no difference in band intensity between control animals and treated animals (Fig. 1), despite large differences in liver iron content (Table 1) and *Hamp* mRNA levels (Fig. 2). These results indicate that total liver Hjv content does not change following manipulation of liver iron levels.

**Table 1:** Effect of experimental protocols on iron status in mice.

Treatment	Hemoglobin	Hematocrit	MCV	Liver Iron	Plasma Iron
	g/l	%	fl	µg/g wet wt.	µmol/l
Control	149.7±4.7	46.2±2.5	45±1	35.7±10.0	22.2±8.9
Iron	149.8±2.0	47,5±1.1	46±1	2165±186	75.5±5.5 *
EPO	170.3±4.9 *	51.9±1.3 *	47±1	25.7±2.7 *	6.1±6.8 *
Control diet	145.8±5.0	40.2±1.1	44±1	82.1±20.7	22.0±3.3
Low iron diet	144.6±5.2	41.0±1.4	42±2	35.2±19.6 *	10.7±2.1 *
Bleeding	63±10	19±3	37±1	14±6 *	8.2±1.1 *

Iron was administered to male C57BL/6 mice as iron polyisomaltosate at 200 mg/kg, erythropoietin (EPO) was injected at 50 U/mouse on days 1–4, mice were sacrificed on day 5. Low iron diet was fed to female C57BL/6 mice for four weeks after weaning, bleeding was performed once weekly (0.6 ml of blood) by retrobulbar puncture in halothane anesthesia for four weeks. Asterisk denotes significant difference from controls (p<0.05, n≥4).

**Figure 3:**
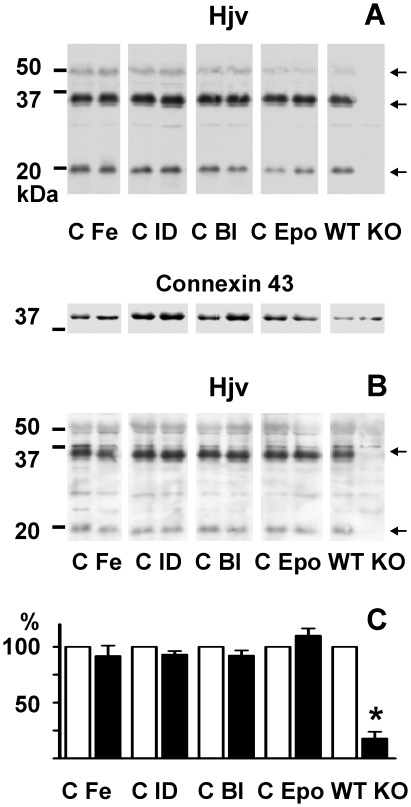
Effect of iron status on mouse liver membrane hemojuvelin. Immunoblots of Hjv protein in liver crude membrane fractions. Panel A: Detection with primary anti-Hjv antibody AF3634. Panel B: Identical samples detected with primary anti-Hjv antibody AF3720. Panel C: Densitometric quantification of the 35 kDa Hjv band. C: Control, Fe: Iron injection (200 mg/kg), Bl: Bleeding (0.6 ml of blood once weekly for 3 weeks), ID: Iron-deficient diet, Epo: Erythropoietin (50 U/day) administration for 4 days, WT: *Hjv*+/+ mice, KO: *Hjv*−/− mice. 60 µg of protein was loaded per lane. Arrows indicate Hjv-specific bands. Connexin 43 was used as loading control. For densitometry, the optical density of control samples was set at 100%, n = 4.

Since hemojuvelin exists in both soluble and membrane-bound forms [27], and since the Bmp6-dependent signaling is postulated to depend on membrane-bound hemojuvelin, we next determined the Hjv protein content in crude membrane fractions obtained by ultracentrifugation. The Hjv signal from the crude membrane fraction was generally stronger than the signal from whole liver homogenate, and an additional weak band at 50 kDa, probably representing the uncleaved membrane-bound Hjv monomer, could be detected in some blots (Fig. 3A). Both the 35 kDa and 20 kDa Hjv-specific bands were readily visualized by another antibody (AF3720) from the same company (Fig. 3B). Comparison of samples from control mice and treated mice showed no significant change in optical density of the major 35 kDa band (Fig. 3C), indicating that membrane Hjv is not significantly altered by iron overload or iron deficiency. To confirm that the manipulation of iron levels does not produce any artificial bands on immunoblots, we also subjected individual *Hjv−/−* mice to similar experimental treatment (iron administration, bleeding, and EPO administration). No additional bands were observed (Figure S1).

**Figure 4:**
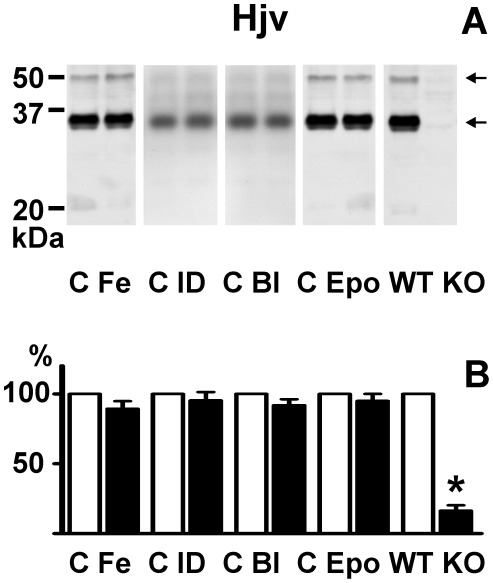
Effect of iron status on mouse liver GPI-bound membrane hemojuvelin. Panel A: Immunoblot of supernatants from Pi-PLC-treated samples. Panel B: Densitometric quantification of the 35 kDa Hjv band. Pi-PLC treatment was performed as described in Materials and Methods. C: Control, Fe: Iron injection (200 mg/kg), Bl: Bleeding (0.6 ml of blood once weekly for 3 weeks), ID: Iron-deficient diet, Epo: Erythropoietin (50 U/day) administration for 4 days, WT: *Hjv*+/+ mice, KO: *Hjv*−/− mice. Arrows indicate Hjv-specific bands. 60 µg of protein was loaded per lane, equal protein loading was verified by staining of the membrane with Amido Black dye. Primary anti-Hjv antibody: AF3634. For densitometry, the optical density of control samples was set at 100%, n = 3.

Hjv is attached to the plasma membrane by a GPI anchor [27], [28]. We have previously demonstrated that cleavage of the GPI-binding enhances the signal from the 35 kDa band, and, in addition, enables reproducible detection of the putative 50 kDa monomer [26]. Therefore, in an additional experiment, liver crude membrane fraction samples were treated with phosphatidylinositol-specific phospholipase C (Pi-PLC). This treatment resulted in the release into the supernatant of two hemojuvelin-specific bands: a major band of 35 kDa, and a weaker band of 50 kDa (Fig. 4A). As noted previously [26], the intensity of the 20 kDa band strongly decreased after Pi-PLC treatment. No changes in the intensities of the 35 kDa band were apparent after iron overload, iron depletion, bleeding or erythropoietin administration (Fig. 4B).

**Figure 5:**
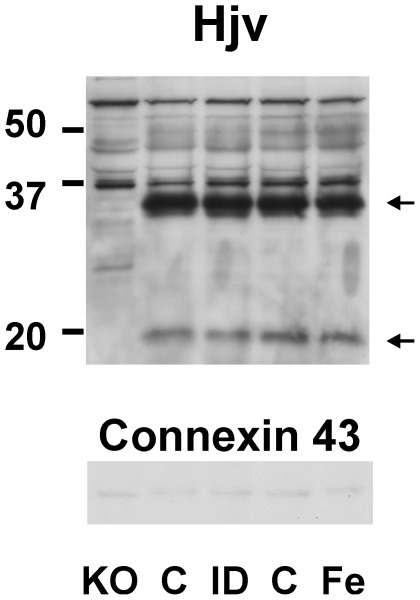
Effect of iron status on mouse liver plasma mebrane hemojuvelin. Immunoblots of hemojuvelin in samples enriched in plasma membrane fraction. Samples were obtained with a commercial plasma membrane extraction kit. KO: *Hjv*−/− mice, C: Control, ID: Iron deficient diet, Fe: Iron injection (200 mg/kg). Arrows indicate Hjv-specific bands. Connexin 43 was used as loading control, 40 µg of protein was loaded per lane. Primary anti-Hjv antibody: AF3720.

It has previously been reported that the amount of Hjv present in the plasma membrane is relatively low compared to total membrane Hjv protein [10]. Therefore, as an attempt to quantify possible changes in plasma membrane Hjv, an additional set of samples was prepared using a commercial plasma membrane protein extraction kit. Total yield from a 150 mg piece of liver was about 90 µg of protein enriched in plasma membrane fraction. Immunoblotting of the liver plasma membrane fraction from iron-overloaded and iron-deficient mice did not show any marked differences between the samples (Fig. 5).

### Manipulation of iron levels does not change membrane hemojuvelin content in rats

**Figure 6:**
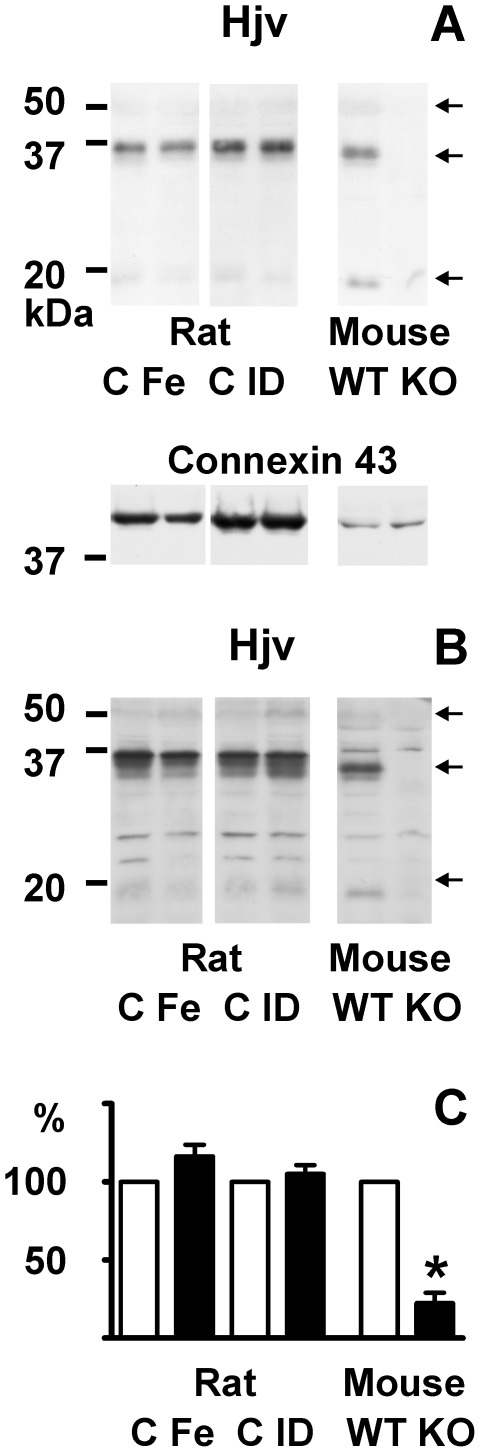
Effect of iron status on rat liver membrane hemojuvelin. Immunoblots of Hjv protein in rat liver crude membrane fractions. Panel A: Detection with primary anti-Hjv antibody AF3634. Panel B: Identical samples detected with primary anti-Hjv antibody AF3720. Panel C: Densitometric quantification of the 35 kDa Hjv band. Liver samples from *Hjv+/+* and *Hjv−/−* mice are included for comparison. C: Control; Fe: Iron injection (1200 mg/kg); ID: Iron-deficient diet. WT: *Hjv*+/+ mice, KO: *Hjv*−/− mice. 60 µg of protein was loaded per lane. Arrows indicate Hjv-specific bands. Connexin 43 was used as loading control. For densitometry, the optical density of control samples was set at 100%, n = 3.

Since the mature mouse Hjv protein shares more than 95% amino acid identity with rat Hjv protein, it was of interest to determine whether the anti-mouse Hjv antibodies could also be used to determine Hjv protein levels in rats. As can be seen in Fig. 6A and B, both antibodies detected a band around 35 kDa in rat liver crude membrane preparations, and a much weaker band at about 20 kDa. Under non-reducing conditions, the antibody detected a single band of about 50 kDa (results not shown), which is in agreement with the proposed composition of the Hjv heterodimer.

Treatment of Wistar rats with iron-dextran increased liver *Hamp* mRNA levels to approximately 230%, while feeding of an iron deficient diet decreased *Hamp* mRNA expression to less than 1% (results not shown). Liver and plasma iron content significantly decreased after feeding of an iron-deficient diet (Table 2). None of the treatments significantly changed the intensity of the major 35 kDa band (Fig. 6C), confirming the recent observation that iron deficiency does not influence rat liver total membrane Hjv protein levels [10]. Overall, the immunoblotting experiments did not demonstrate any substantial changes in liver hemojuvelin protein content after manipulation of liver iron levels.

**Table 2:** Effect of experimental protocols on iron status in rats.

Treatment	Hemoglobin	Hematocrit	MCV	Liver Iron	Plasma Iron
	g/l	%	fl	µg/g wet wt.	µmol/l
Control	145.3±2.5	44.7±1.0	60±1	55±13.0	24.7±2.9
Iron	145.7±3.1	45.3±2.5	59±1	3340±520*	62.3±3.7*
Control diet	140.3±1.3	42.0±0.3	59±1	138±87	48.5±7.7
Low iron diet	53.0±5.4	16.3±2.1	41±1	19±2 *	6.0±3.8*

Iron was administered to male Wistar rats as iron-dextran in three weekly injections (100 mg iron/rat), total administered dose was 300 mg iron/rat. Low iron diet was fed to female Wistar rats for four weeks after weaning. Asterisk denotes significant difference from controls (p<0.05, n = 3).

### Iron deficiency does not result in detectable changes of mouse plasma hemojuvelin

**Figure 7:**
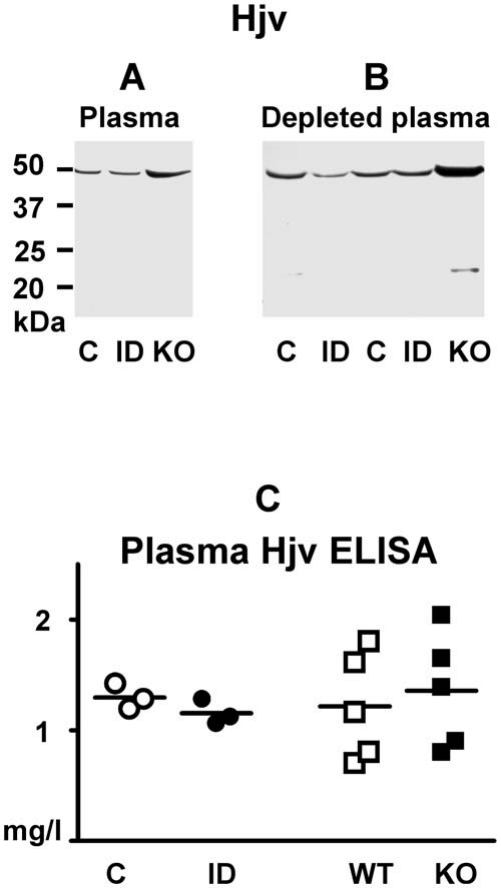
Determination of mouse plasma hemojuvelin by immunoblot and ELISA. Panel A: Immunoblot of untreated mouse plasma (80 µg of protein per lane). Panel B: Immunoblot of albumin-depleted mouse plasma (60 µg of protein per lane). Primary anti-Hjv antibody: AF3720. Panel C: Plasma Hjv determination by a commercial ELISA kit. C: Control diet; ID: Iron-deficient diet; WT: *Hjv*+/+ mice; KO: *Hjv*−/− mice.

Soluble Hjv has been postulated to function as a negative regulator of *Hamp* gene expression [27]. According to this concept, soluble Hjv levels would be expected to increase in iron deficiency. We therefore attempted to detect soluble Hjv by immunoblot, in both whole and albumin-depleted plasma, as well as by a commercially available ELISA kit. By immunoblotting of mouse plasma, no differences were detected between animals fed normal diet or iron-deficient diet for two months, despite a marked decrease of liver *Hamp* mRNA in the latter (Fig. 7A). Similar results were obtained in rats (results not shown). No Hjv-specific band could be identified. Removal of the majority of albumin and IgG from mouse plasma samples by an immunoaffinity depletion kit enhanced the visualization of less abundant proteins; however, comparison of samples from *Hjv*+/+ and *Hjv*−/− mice again did not detect any Hjv-specific bands (Fig. 7B). In both untreated and albumin-depleted plasma, a strong non-specific band was apparent at about 50 kDa.

Next, it was attempted to quantify plasma Hjv by a commercial mouse Hjv ELISA kit. Although the assay generated a linear calibration curve (y = 8.7291x−0.3426, R^2^ = 0.9955), and the obtained values were well within the calibration curve range, no significant difference was seen between three pairs of plasma from mice fed control diet or iron-deficient diet (Fig. 7C). Intriguingly, comparison of five pairs of plasma samples from *Hjv*+/+ and *Hjv*−/− mice did not show any difference between the two groups (Fig. 7C), despite an almost complete absence of *Hjv* mRNA in the liver, muscle and heart of *Hjv*−/− animals [11].

### Erythropoietin treatment influences Bmp-dependent signaling

To confirm the effect of the various experimental treatments on iron homeostasis, liver *Hamp* mRNA levels were determined by real-time PCR. As expected, iron treatment increased liver *Hamp* expression, while feeding of an iron-deficient diet, repeated bleeding or administration of erythropoietin dramatically decreased *Hamp* mRNA content (Fig. 2). Iron treatment increased, and repeated bleeding decreased, *Bmp6* mRNA, while *Tmprss6* mRNA was unchanged by manipulation of hepatic iron (Fig. 2).

The effect of the various treatments on Bmp-dependent signaling was examined by monitoring hepatic *Id1* mRNA levels. *Id1* is a well-recognized target of Bmp6 signaling [9], and the determination of *Id1* mRNA is in some cases more sensitive [29] than the determination of phosphorylated Smad proteins, which is traditionally [5] used for the monitoring of Bmp signaling pathway. As expected, iron administration markedly increased hepatic *Id1* mRNA levels, while iron-deficient diet or repeated bleeding resulted in a statistically significant decrease. Interestingly, EPO treatment also decreased liver *Id1* mRNA content (Fig. 2), suggesting that the the Bmp/Hjv pathway could participate in the well-documented downregulation of *Hamp* gene expression by accelerated erythropoiesis. Liver *Id1* mRNA levels were slightly decreased in *Hjv*−/− mice; however, in contrast to results described in *Bmp6*−/− mice [7], the decrease did not reach statistical significance, indicating that *Id1* gene transcription is less sensitive to the loss of Hjv than *Hamp* gene transcription.

### High dose erythropoietin decreases liver iron and plasma iron

Although EPO administration is known to decrease *Hamp* expression [30], the exact mechanism by which accelerated erythropoiesis influences *Hamp* transcription is still unclear [1], [2], [3]. It has been proposed that the EPO-induced decrease of plasma iron could be an important contributing factor [31]. To confirm the reported effect of EPO on iron metabolism [31], we examined plasma iron content and liver non-heme iron content after four days of EPO treatment. As can be seen in Table 1, daily administration of a high dose (50 U) of EPO markedly decreased plasma iron content, and, in addition, also caused a decrease in liver iron content. These data confirm that the traditional experimental protocol (50 U of EPO for several days), often used to accelerate erythropoiesis in iron metabolism studies [30], [31], [32], causes significant mobilization of storage iron from the liver.

## Discussion

The aim of the study was to determine the effect of altered iron homeostasis on Bmp-dependent signaling, with particular emphasis on hepatic hemojuvelin protein levels.

Hjv is well recognized as a major component of the Bmp/Hjv signaling pathway, which plays a crucial role in the regulation of *Hamp* gene expression by iron. It has already been demonstrated that experimental manipulation of iron levels *in vivo* does not change *Hjv* mRNA [33]; however, there is little information about the effect of iron overload or iron deficiency on Hjv protein levels. In *in vitro* experiments with transfected cells, there was no apparent effect of iron treatment on cellular Hjv [34], [35]. On the other hand, the recent demonstration that matriptase-2 cleaves Hjv [22] suggests that the amount of membrane Hjv protein could be an important determinant of hepcidin expression. In one study with a hepatic cell line, matriptase-2 protein levels have been reported to increase in hypoxia [25], resulting in lower Hjv levels at the plasma membrane.

In the presented experiments, we attempted to determine the effect of iron status on liver Hjv protein levels in whole liver homogenates and total liver membrane fractions. Using commercial anti-Hjv antibodies, and Hjv-deficient mice as negative control, we identified two Hjv-specific bands in liver homogenates, very probably representing two components of a membrane-bound Hjv heterodimer. Interestingly, no apparent changes in the intensity of these bands were observed following iron overload or iron depletion. The same results were obtained using liver crude membrane fraction, again demonstrating that manipulation of iron levels does not change the total amount of membrane Hjv protein. In addition, no changes in Hjv band intensities were observed following iron treatment, iron depletion or erythropoietin administration in a series of samples containing liver proteins bound to the membrane by a GPI anchor.

Since it has been reported that plasma membrane Hjv represents only a small fraction of total membrane Hjv [10], it was also attempted to determine Hjv protein in samples enriched in the plasma membrane fraction. The resulting pattern of Hjv bands in this plasma membrane-enriched sample, obtained by a commercial plasma membrane protein extraction kit, was similar to the pattern obtained from crude membrane preparation, and no apparent changes were observed following iron overload or iron depletion.

In conclusion, using five experimental sources of Hjv protein (mouse whole liver homogenate, crude membrane fraction, GPI anchor-bound protein fraction, plasma membrane-enriched fraction and rat liver crude membrane fraction), we were unable to detect changes in Hjv content following iron overload or iron depletion. These data suggest that, *in vivo*, dramatic changes in hepcidin transcription can occur without noticeable changes in hemojuvelin protein.

Although the results apparently do not support the concept that matriptase-2 controls *Hamp* gene expression by modulating membrane Hjv levels, there are important caveats to this conclusion. First, the actual amount of plasma membrane Hjv in contact with the Bmp6 receptor complex might be too small in comparison with total membrane Hjv present in the crude membrane preparation, or even in the plasma-membrane-enriched fraction. Also, it is conceivable that the antibodies used in the study do not detect all Hjv fractions present in the cell, particularly as Hjv is known to undergo complex processing [36], [37]. Nevertheless, together with our recent demonstration that liver Hjv protein actually decreases in *Tmprss6−/−* mice [26], the presented results could point to possible differences between the *in vitro* and *in vivo* mode of action of matriptase-2, suggesting that matriptase-2 could have other potential targets in addition to hemojuvelin.

Theoretically, the interaction between Hjv and the Bmp6 receptor complex could be modulated by soluble Hjv [27], possibly originating from extrahepatic tissues [34]. It was therefore also attempted to detect an increase in plasma Hjv levels after iron depletion. Although both antibodies reproducibly visualize Hjv-specific bands in liver samples, Hjv-specific bands in mouse and rat plasma or in albumin-depleted mouse plasma were not seen. Moreover, using a recently introduced commercial mouse Hjv ELISA kit, we did not detect any differences in plasma Hjv content between samples obtained from *Hjv*+/+ and *Hjv*−/− mice. Thus, in our mouse and rat models of iron depletion, we were unable to confirm the role of soluble Hjv in *Hamp* gene regulation *in vivo*. It should be noted that the release of soluble Hjv does not represent the sole mechanism responsible for *Hamp* gene downregulation, since repeated phlebotomies or erythropoietin administration decrease liver *Hamp* mRNA even in *Hjv*−/− mice [38]. Obviously, more research effort will be necessary to firmly establish the tissue(s) of origin, as well as the exact physiological role of plasma hemojuvelin.

In the presented experiments, it was attempted to manipulate Hjv protein levels by three stimuli – by iron overload, by iron deficiency, and also by stimulation of erythropoiesis. As expected, both iron deficiency and EPO administration caused a dramatic drop in *Hamp* mRNA. The “erythroid regulator” pathway, which controls hepcidin transcription following changes in erythropoietic activity, constitutes the least understood part of hepcidin gene regulation [2]. Several candidates for the molecules linking accelerated erythropoiesis in the bone marrow and spleen with liver *Hamp* gene expression have been proposed [39], but their role is probably restricted to pathological conditions such as thalassemias [40]. Results from the presented experiments demonstrate that the dramatic decrease in *Hamp* transcription following EPO treatment is not mediated by changes in Hjv protein. However, treatment with erythropoietin decreased hepatic *Id1* mRNA. *Id1* is a sensitive indicator of Bmp6-dependent signaling [9], and the decrease in *Id1* mRNA after EPO treatment suggests that, under physiological conditions, the Bmp/Hjv pathway could participate in EPO-dependent downregulation of *Hamp* transcription. These results agree with the observation made by Huang *et al.* [41], who reported an EPO-mediated decrease in iron-induced levels of phosphorylated Smad proteins. Thus, there apparently is some cross talk between the Bmp/Hjv signaling pathway and the erythropoiesis-regulated pathway, despite the fact that mice lacking functional Hjv protein are still able to downregulate *Hamp* mRNA following EPO administration [38]. It is worth noting that the dose of EPO used in this and most other experiments [30], [31], [32] is relatively high, and causes substantial drop in plasma iron content, as well as a decrease in liver iron content. It is therefore possible that the effect of EPO on hepcidin transcription, generally attributed to some soluble circulating factor produced during erythropoiesis [31], is in fact mainly caused by the EPO-induced flow of iron from tissue stores and plasma to the erythroid compartment. Further studies will be needed to clarify the role of accelerated erythropoiesis, and particularly ineffective erythropoiesis, on hepcidin downregulation.

In conclusion, the presented study demonstrated that high doses of EPO decrease liver *Id1* mRNA, suggesting that accelerated erythropoiesis influences signaling through the bone morphogenetic protein receptors. As main result, we consistently found unchanged hemojuvelin protein levels following various manipulations of liver iron levels. This indicates that the total amount of hepatocyte membrane hemojuvelin is not a critical determinant of hepatic hepcidin gene expression *in vivo*.

## Materials and Methods

### Ethics Statement

All animal experiments were approved by the Ethics Committee of the First Faculty of Medicine in Prague, protocol number MSM 0021620806. Phlebotomies were performed under halothane anesthesia.

### Animals and Treatment

8–10 week old male C57BL/6 mice were used for iron and EPO administration, female C57BL/6 mice of the same age were used for experiments with iron-deficient diet and repeated bleeding. 0.6 ml of blood was withdrawn once weekly by retrobulbar puncture for three weeks, and bled mice were kept on an iron deficient diet for the duration of the experiment. For experiments with dietary-induced iron deficiency, female mice aged 4 weeks were fed an iron-deficient diet (Altromin GmbH, Lage, Germany) for the next four weeks, or, in studies concerning plasma Hjv, for eight weeks. Iron was administered as iron polyisomaltosate (Ferrum Lek, Lek Ljubljana, Slovenia) at 200 mg/kg by intraperitoneal injection and mice were sacrificed one week later. EPO (NeoRecormon®, Roche) was administered once daily at 50 U/mouse for four days, mice were sacrificed 24 hours after last EPO administration.

Male outbred Wistar rats (250 g) were administered three doses of iron-dextran (Sigma-Aldrich, 100 mg iron/week) over three weeks, and sacrificed one week later. Female Wistar rats (approximately 50 g) were placed on an iron deficient diet for four weeks immediately after weaning, body weight at the time of sacrifice was approximately 160 g.

Male *Hjv*−/− mice, age 3–4 months, were a generous gift from Prof. Silvia Arber, Basel, Switzerland [11]. Real-time PCR of tissue cDNA samples from *Hjv−/−* mice confirmed absence of *Hjv* cDNA corresponding to coding exon 2 mRNA in liver, muscle and myocardium [11], while cDNA corresponding to exon 4 was dramatically decreased (results not shown).

### Immunoblotting

Anti-Hjv antibodies (AF3634 and AF3720) were purchased from R&D Systems (Minneapolis, MN, USA), anti-Gapdh antibody (G9545) from Sigma Aldrich, and anti-connexin 43 antibody from Cell Signaling Technology (Danvers, MA, USA). Secondary antibodies (705-036-147 and 713-036-137) were obtained from Jackson ImmunoResearch Europe, UK.

For Hjv protein determination in whole tissue homogenates, tissue samples were homogenized (5×10 sec) with an Ultra Turrax homogenizer in 5 volumes of Ripa buffer (Sigma Aldrich) with a protease inhibitor mix (Roche Diagnostics, Mannheim, Germany) and centrifuged for 15 min at 6000 g. 80 µg of supernatant protein was loaded on pre-cast 4–20% Tris-Glycine gel (Invitrogen, Carlsbad, CA, USA). For liver membrane Hjv protein determinations, liver was homogenized in 5 volumes of 0.25 mM sucrose, pH 7.6, containing 1 mM EDTA and protease inhibitors. Homogenate was centrifuged for 15 min at 6000 g, and membranes were obtained by ultracentrifugation at 80 000 g for 55 min. 60 µg of the membrane proteins were separated by SDS electrophoresis under reducing conditions (2% β-mercaptoethanol) on 12% polyacrylamide gels.

For phosphatidylinositol-specific phospholipase C cleavage of the GPI anchor, liver membrane samples (4 mg of protein) were treated with Pi-PLC (Sigma-Aldrich, 100 mU) at 37°C for 3 hours and subsequently recentrifuged at 80 000 g for 45 min. 60 µg of supernatant protein was loaded per well.

Plasma membrane proteins were extracted by Plasma Membrane Protein Extraction Kit (BioVision Research Products, CA, USA); starting amount was 150 mg of mouse liver. After collection of the total cellular membrane protein pellet, plasma membrane proteins were purified by adding 600 µl of the Upper Phase Solution and further separated according to the manufacturer protocol. The plasma membrane pellet was resuspended in 40 µl of 0.5% Triton X-100 in phosphate-buffered saline; for electrophoresis, 40 µg of protein was loaded per well.

Mouse and rat plasma was diluted 1∶2 with β-mercaptoethanol-containing loading buffer and loaded at 80 µg per well. For albumin and IgG depletion of mouse plasma, Sigma ProteoPrep® kit was used according to manufacturer instructions; 60 µg of depleted plasma was loaded per lane.

Proteins were blotted on a PVDF membrane, blots were blocked with 5% skimmed milk for one hour and incubated overnight with a solution of the primary antibody in 5% milk. Primary antibody dilutions were 1∶500 for Hjv and connexin 43, and 1∶100 000 for Gapdh. After washing, blots were incubated with a 1∶20 000 solution of the secondary antibody and proteins were visualized by chemiluminiscence. Densitometry of the 35 kDa bands was performed on a Biorad GS 800 scanner; for statistical analysis, the optical density of the control sample was set as 100%.

### Plasma Hjv determination by ELISA

A commercial kit (E91995Mu, Uscn Life Sciences Inc) was used for mouse plasma Hjv determination. Heparinised blood was collected by retrobulbar puncture, and plasma was obtained by centifugation at 1000 g for 15 min. ELISA was performed immediately after separation of plasma on 1∶200 diluted samples.

### Real-Time PCR and Iron Determinations

RNA isolation and real-time PCR was performed as described previously [38], *Actb* primers were GACATGGAGAAGATCTGGCA (forward) and GGTCTTTACGGATGTCAACG (reverse); *Id1* primers were CGAGGTGGTACTTGGTCTGTC (forward) and CTGCAGGTCCCTGATGTAGTC (reverse). Plasma iron was determined spectrophotometrically by BioLaTest Fe 100 kit (PLIVA-Lachema Diagnostika, Brno, Czech Republic), liver iron was determined according to Torrance and Bothwell [42]. Blood counts were determined on Bayer Advia 60 system.

### Statistics

Statistical analysis of real-time PCR results was performed using the Mann-Whitney test, iron metabolism parameters and densitometry results were analyzed by unpaired t-test.

## Supporting Information

Figure S1 Hjv immunoblot in iron-overloaded and iron-depleted *Hjv*−/− mice. Male *Hjv*−/− mice were subjected to similar experimental treatments as C57BL/6 mice. WT: *Hjv+/+* mice, KO: *Hjv−/−* mice, C: Control, Fe: Iron injection (200 mg/kg), Bl: Bleeding (0.6 ml of blood once weekly for 3 weeks), Epo: Erythropoietin (50 U/day) administration for 4 days. Primary anti-Hjv antibody: AF3720. 60 µg of protein was loaded per lane. Connexin 43 was used as loading control. Arrows indicate Hjv-specific bands.
(TIF)
