# Supplementary figures and images for: Effect of Iron Overload and Iron Deficiency on Liver Hemojuvelin Protein
Source: PLoS One. 2012 May 18;7(5):e37391. doi: 10.1371/journal.pone.0037391 (PMC3356351; doi:10.1371/journal.pone.0037391)

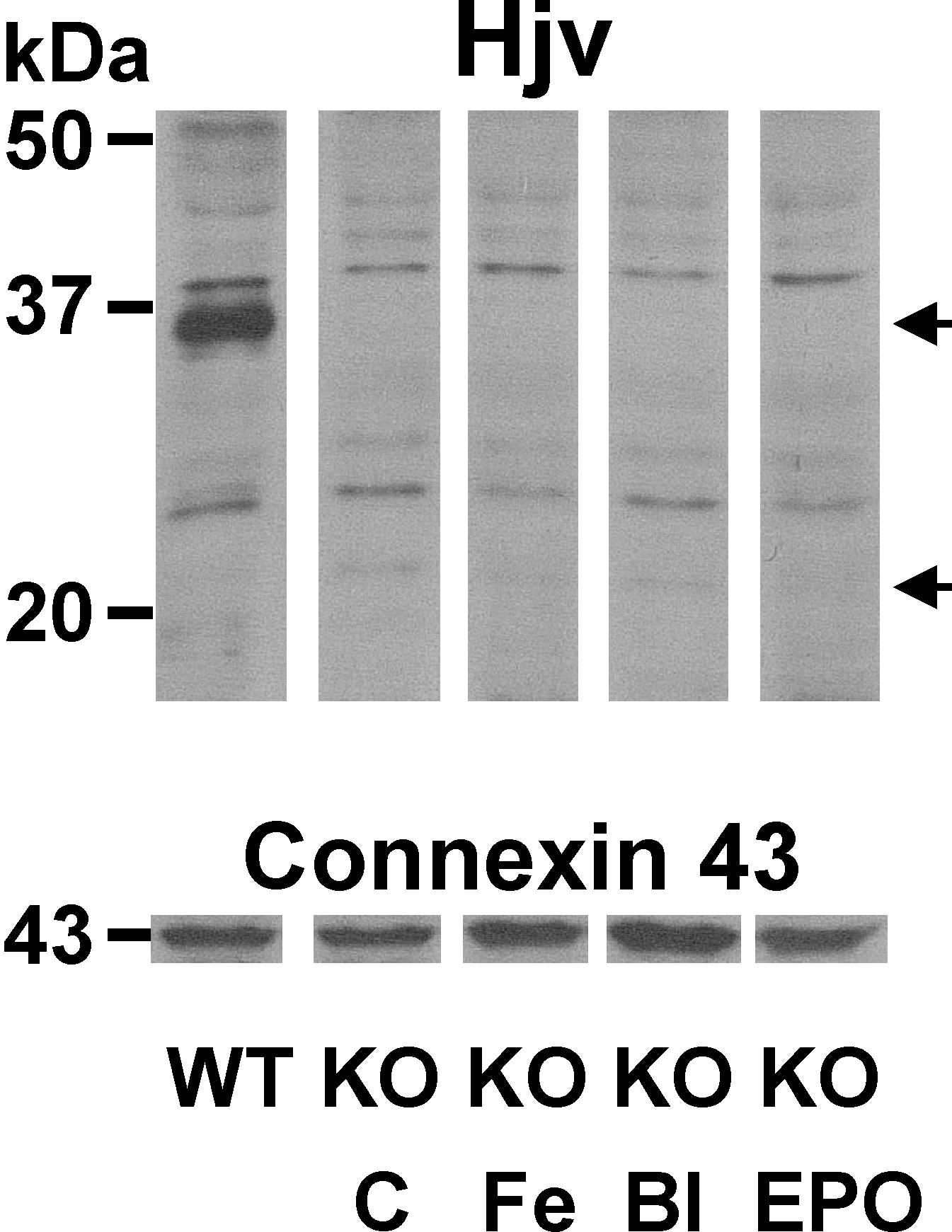

Supplement: Figure S1 — Hjv immunoblot in iron-overloaded and iron-depleted Hjv−/− mice. Male Hjv−/− mice were subjected to similar experimental treatments as C57BL/6 mice. WT: Hjv+/+ mice, KO: Hjv−/− mice, C: Control, Fe: Iron injection (200 mg/kg), Bl: Bleeding (0.6 ml of blood once weekly for 3 weeks), Epo: Erythropoietin (50 U/day) administration for 4 days. Primary anti-Hjv antibody: AF3720. 60 µg of protein was loaded per lane. Connexin 43 was used as loading control. Arrows indicate Hjv-specific bands. (TIF) [file pone.0037391.s001.tif]
